# Characterization of glucose metabolism in breast cancer to guide clinical therapy

**DOI:** 10.3389/fsurg.2022.973410

**Published:** 2022-09-19

**Authors:** Yingying Mei, Lantao Zhao, Man Jiang, Fangfang Yang, Xiaochun Zhang, Yizhen Jia, Na Zhou

**Affiliations:** ^1^ Precision Medicine Center of Oncology, The Affiliated Hospital of Qingdao University, Qingdao University, Qingdao, China; ^2^ Department of Anesthesiology, The Affiliated Hospital of Qingdao University, Qingdao, China; ^3^ Core Laboratory, The University of Hong Kong-Shenzhen Hospital, Shenzhen, China

**Keywords:** breast cancer, glucose metabolism, prognosis, immune infiltration, drug sensitivity

## Abstract

**Background:**

Breast cancer (BRCA) ranks as a leading cause of cancer death in women worldwide. Glucose metabolism is a noticeable characteristic of the occurrence of malignant tumors. In this study, we aimed to construct a novel glycometabolism-related gene (GRG) signature to predict overall survival (OS), immune infiltration and therapeutic response in BRCA patients.

**Materials and methods:**

The mRNA sequencing and corresponding clinical data of BRCA patients were obtained from public cohorts. Lasso regression was applied to establish a GRG signature. The immune infiltration was evaluated with the ESTIMATE and CIBERSORT algorithms. The drug sensitivity was estimated using the value of IC50, and further forecasted the therapeutic response of each patient. The candidate target was selected in Cytoscape. A nomogram was constructed *via* the R package of “rms”.

**Results:**

We constructed a six-GRG signature based on CACNA1H, CHPF, IRS2, NT5E, SDC1 and ATP6AP1, and the high-risk patients were correlated with poorer OS (*P* = 2.515 × 10^−7^). M2 macrophage infiltration was considerably superior in high-risk patients, and CD8^+^ T cell infiltration was significantly higher in low-risk patients. Additionally, the high-risk group was more sensitive to Lapatinib. Fortunately, SDC1 was recognized as candidate target and patients had a better OS in the low-SDC1 group. A nomogram integrating the GRG signature was developed, and calibration curves were consistent between the actual and predicted OS.

**Conclusions:**

We identified a novel GRG signature complementing the present understanding of the targeted therapy and immune biomarker in breast cancer. The GRGs may provide fresh insights for individualized management of BRCA patients.

## Introduction

The International Agency for Research on Cancer (IARC) recently released its latest estimates of the global cancer burden, with breast cancer defined as the number one cancer in 2020 compared to 2018 (1). Breast cancer is an important reason for cancer-associated deaths around the world, and is currently the first killer seriously threatening women's health. It is located in the first place of incidence and the second place of mortality in female tumors (1, 2). Compared with early breast cancer, the situation of advanced breast cancer is more serious (3). The results of clinical studies have shown that 1 in 10 new patients is diagnosed with advanced breast cancer, and 20% to 30% of patients with early breast cancer will deteriorate to advanced breast cancer (4). Among them, the median OS in advanced BRCA patients is only 2 to 3 years, 5 years survival rate is only about 25% (5). Because of its high aggressiveness, targeted therapy is an interesting area of research to find non-endocrine therapies for breast cancer. Despite preliminary advances in targeted therapy, drug resistance is still a vital clinical challenge in the failure of current therapy in breast cancer. Therefore, the relatively optimal targeted therapies require further research in BRCA patients (6).

Energy metabolism reprogramming is designed to accelerate tumor cell growth and proliferation by regulating the process of glucose metabolism, which has been considered to be a new sign of cancer (7, 8). Glucose metabolism is the main pathway for tumor cells to obtain ATP, including glycolysis and oxidative phosphorylation (9, 10). Tumor cells generally have the characteristics of unchecked proliferation, meaning that they need abundant glucose to provide energy (11). In aerobic conditions, normal cells obtain energy through mitochondrial oxidative phosphorylation. While in the absence of oxygen, cells obtain energy through glycolysis rather than mitochondrial metabolism of consuming oxygen (12, 13). In the 1920s, Warburg found that even under aerobic conditions, tumor cells were more energetic in glycolysis to obtain ATP for metabolic activities (14). This abnormal phenomenon of glucose metabolism was called aerobic glycolysis, also known as Warburg effect (14). Hence, understanding the abnormal energy metabolism of tumor cells is of great significance in finding new anti-tumor therapies.

With the development of High-Throughput Sequencing technology, genome databases of various diseases have been established successively, enabling us to have a deeper comprehending of genome variations (15, 16). Some clinical trials have noticed that patients with a similar extent of progression may show different outcomes and endings (17). Therefore, it is necessary to search for effective biomarkers to assess and identify potential breast cancer patients who are in high-risk circumstances. Researchers have explored the influence of polygenic characteristics on tumors, prompting that it can be used to assess prognosis and identify patients at potential high-risk of malignancy (18). Consistently, polygenic prognostic characteristics of primary tumors may guide more specific treatment strategies (19).

## Materials and methods

### Data source

The mRNA sequencing data and corresponding clinical features of BRCA patients were derived from TCGA (https://portal.gdc.cancer.gov/) for training data. The TCGA cohort consists of 1,109 BRCA samples and 113 adjacent normal samples, among which 1,076 patients had complete follow-up data, whose clinical information included Age, Gender, AJCC TNM, Stage and Vital status. The clinicopathological features were shown in Table 1. The validation data was obtained from ICGC (https://dcc.icgc.org/) and GSE7390 cohorts (https://www.ncbi.nlm.nih.gov/geo/). The TCGA, ICGC and GEO databases were open access and publicly available, and the study followed data access policies and published guidelines (20). Then, the gene sets of main hallmarks of glycometabolism, including glycolysis and oxidative phosphorylation, were retrieved from the Molecular Signatures Database (MsigDB, http://www.gsea-msigdb.org/gsea/msigdb) to obtain the glycometabolism-related genes (GRGs) (21).

**Table 1:** Clinicopathological parameters of patients with BRCA in the study.

Clinical characteristic	TCGA cohort (*N* = 1,076) *N* (%)	ICGC cohort (*N* = 1,039) *N* (%)	GSE7390 cohort (*N* = 198) *N* (%)
**Age**			
≦65	773 (71.84)	739 (71.13)	198 (100)
>65	303 (28.16)	300 (28.87)	0 (0)
**Gender**			
Male	12 (1.12)	12 (1.15)	–
Female	1,064 (98.88)	1,027 (98.85)	–
**Stage**			
I	183 (17.01)	–	–
II	608 (56.51)	–	–
III	242 (22.49)	–	–
IV	20 (1.86)	–	–
X	12 (1.12)	–	–
unknow	11 (1.01)	–	–
**AJCC T**			
T1	281 (26.12)	–	–
T2	621 (57.71)	–	–
T3	133 (12.36)	–	–
T4	38 (3.53)	–	–
TX	3 (0.28)	–	–
**AJCC N**			
N0	504 (46.84)	–	–
N1	361 (33.55)	–	–
N2	120 (11.15)	–	–
N3	74 (6.88)	–	–
NX	17 (1.58)	–	–
**AJCC M**			
M0	895 (83.18)	–	–
M1	22 (2.04)	–	–
MX	159 (14.78)	–	–
**Vital status**			
Alive	928 (86.25)	935 (89.99)	142 (71.72)
Dead	148 (13.75)	104 (10.01)	56 (28.28)

Abbreviations: T, Tumor; N, Node (regional lymph node); M, Metastasis.

### Identification of GRG candidates

The Log2 normalization was performed for each gene in the genomic expression spectrum. The “Limma” R package was performed for differential analysis of mRNA expression data to obtain the differentially expressed genes (DEGs) related to glucose metabolism between breast cancer tissues and normal breast tissues (False Discovery Rate (FDR) < 0.05, |Log 2 Fold Change (Log FC)| > 1) (22). DEGs were related to the prognosis of BRCA patients *via* univariable Cox analysis (*P* < 0.05). The “VennDiagram” package of R software was performed to obtain the shared GRGs of the DEGs and the Prognostic genes, and called as GRG candidates. Then, Gene ontology (GO) and Kyoto Encyclopedia of Genes and Genomes (KEGG) pathway enrichment analyses were performed on candidate genes to identify the major biological features and cell functional pathways by the R package of “clusterprofiler” (23).

### Construction and validation of a GRG signature

Firstly, the least absolute shrinkage and selection operator (LASSO) regression was performed with “Glmnet” package to further narrow down the number of candidate genes (24). Then, the R package of “Survival” was used for multivariate Cox regression analysis to determine the best weighting coefficient of candidate GRGs. The GRG signature contained all the prognostic-related GRGs which are differentially expressed. The expression levels of candidate genes were linearly combined with the corresponding regression coefficients of multivariate Cox regression analysis, and the risk score of each patient was calculated using the following formula:

$$\mathrm{Risk}\;\mathrm{score}=\sum_{i=1}^{n}\mathrm{Coef}\;*\;\mathrm{Exp}\;$$

(Coef is the regression coefficient of GRG candidates in multivariate Cox regression, Exp is the expression level of GRG candidates, and *n* is the number of GRG candidates, Table 2).

**Table 2:** Details of candidates for constructing a GRG signature.

Gene	Ensemble ID	Location	HR (95% CI)	Coefficient	*P* value
CACNA1H	ENSG00000196557.9	chr16: 1,153,241–1,221,771	1.1735 (1.0517–1.3094)	0.1281	0.0042
CHPF	ENSG00000123989.12	chr2: 219,538,947–219,543,787	1.2508 (1.0012–1.5626)	0.0016	0.0488
IRS2	ENSG00000185950.8	chr13:109,752,698–109,786,568	0.8202 (0.6860–0.9807)	−0.1299	0.0297
NT5E	ENSG00000135318.10	chr6: 85,449,584–85,495,791	1.2326 (1.0082–1.5070)	0.2311	0.0414
SDC1	ENSG00000115884.9	chr2: 20,200,797–20,225,433	1.2257 (1.0528–1.4270)	0.1604	0.0087
ATP6AP1	ENSG00000071553.15	chrX: 154,428,632–154,436,516	1.3678 (1.0445–1.7912)	0.2888	0.0228

Abbreviation: HR, Hazard ratio.

Furthermore, the GRG signature was applied to generate the risk score for all BRCA patients including TCGA training cohort, ICGC and GSE7390 validated cohorts, and they were divided into high-risk and low-risk groups based on the median risk score as the cut-off value. The principal component analysis (PCA) was performed *via* “*t*-SNE” and “ggplot2” packages in order to confirm the accuracy of grouping in the risk prognostic model (25). ROC and Kaplan–Meier curves were applied to evaluate the performance of the GRG signature. Then, univariate and multivariate Cox regression analyses were used to estimate the independent prognostic contribution of the risk score of GRGs and other clinical characteristics.

### Relationship between risk score of GRG signature and clinical features

Each patient's risk score was combined with their clinical features based on their sample ID. We explored the relationship between risk score and clinical features, including age, gender, AJCC TNM and stage, with the help of the “limma” R package.

### Analysis of tumor microenvironment and GRG signature

We calculated the infiltration of immune cells and stromal cells in tissues of BRCA patients who were in GRG signature, and categorized them as tumor microenvironment (TME) scores, including immune score and stromal score. The potential correlation between risk score and TME score was explored by ESTIMATE algorithm (26). The 22 kinds of tumor-infiltrating immune cells and 13 kinds of immune related function between high-risk and low-risk groups were computed by CIBERSORT algorithm and “GSVA” R package to further explore the immune infiltration associated with our GRG signature (27). Stemness scores containing DNAss and RNAss were analyzed to understand the expression of stemness-related markers in GRG signature by “ggplot” and “ggExtra” package.

### Comparison of antineoplastic therapy between the low-risk and high-risk groups

The sensitivity of each patient to chemotherapy and targeted drugs was estimated using the value of IC50 which was quantified *via* the R package of “pRRophetic” (28). An important indicator of drug effectiveness is half-maximal inhibitory concentration (IC50), where a lower IC50 indicates a high antitumor potential. Tumor mutational burden (TMB) is an emerging biomarker to predict the therapeutic response of immune checkpoint inhibitors (29). We obtained somatic mutation data with BRCA patients from the TCGA database, and the TMB was calculated. The correlation and survival analyses between risk score and TMB were explored to predict effectiveness of immunotherapy.

### Construction of PPI network and identify hub gene

Firstly, we screened for differentially expressed genes between high and low risk groups *via* the “limma” R package (FDR < 0.05, |Log FC| > 1), and which was used to construct the protein-protein interaction (PPI) network in the STRING database (https://string-db.org) (30). Secondly, the PPI network was imported into the Cytoscape (version 3.9.0). Finally, the Hub gene with the highest Degree value was selected in Cytoscape using the cytoHubba plugin for subsequent analysis (31).

### Establishment and validation of a predictive nomogram

A nomogram integrating the GRG signature, gender, age and stage for predicting OS was constructed in TCGA cohort *via* the R package of “rms” (32). Additionally, we plotted calibration curves (33), ROC curves and Cox regression analysis to examine how accurate the nomogram is at forecasting the future health of the patients for the OS probability at 1-, 5- and 10- years.

### Immunohistochemistry of GRG candidates

Immunohistochemistry images of these six GRG candidates were obtained from the Human Protein Atlas (https://www.proteinatlas.org/).

### Statistical analysis

All statistical analyses were performed by R software (version 4.0.3, https://www.r-project.org/) and Perl software (version 5.30.0–64bit, https://stawberryperl.com/). Survival curves between groups were drawn by the Kaplan–Meier method and the Log-rank test. Lasso and multivariate Cox regression analyses were used to calculate regression coefficients and establish a risk prognostic model. A predictive nomogram was established based on the risk score of GRGs and clinical features. Spearman's correlation analysis was used to describe the correlation between variables. All statistics were two-sided tests, and *P* < 0.05 was defined as a statistically significant difference.

## Results

### Identification of GRG candidates

**Figure 1:**
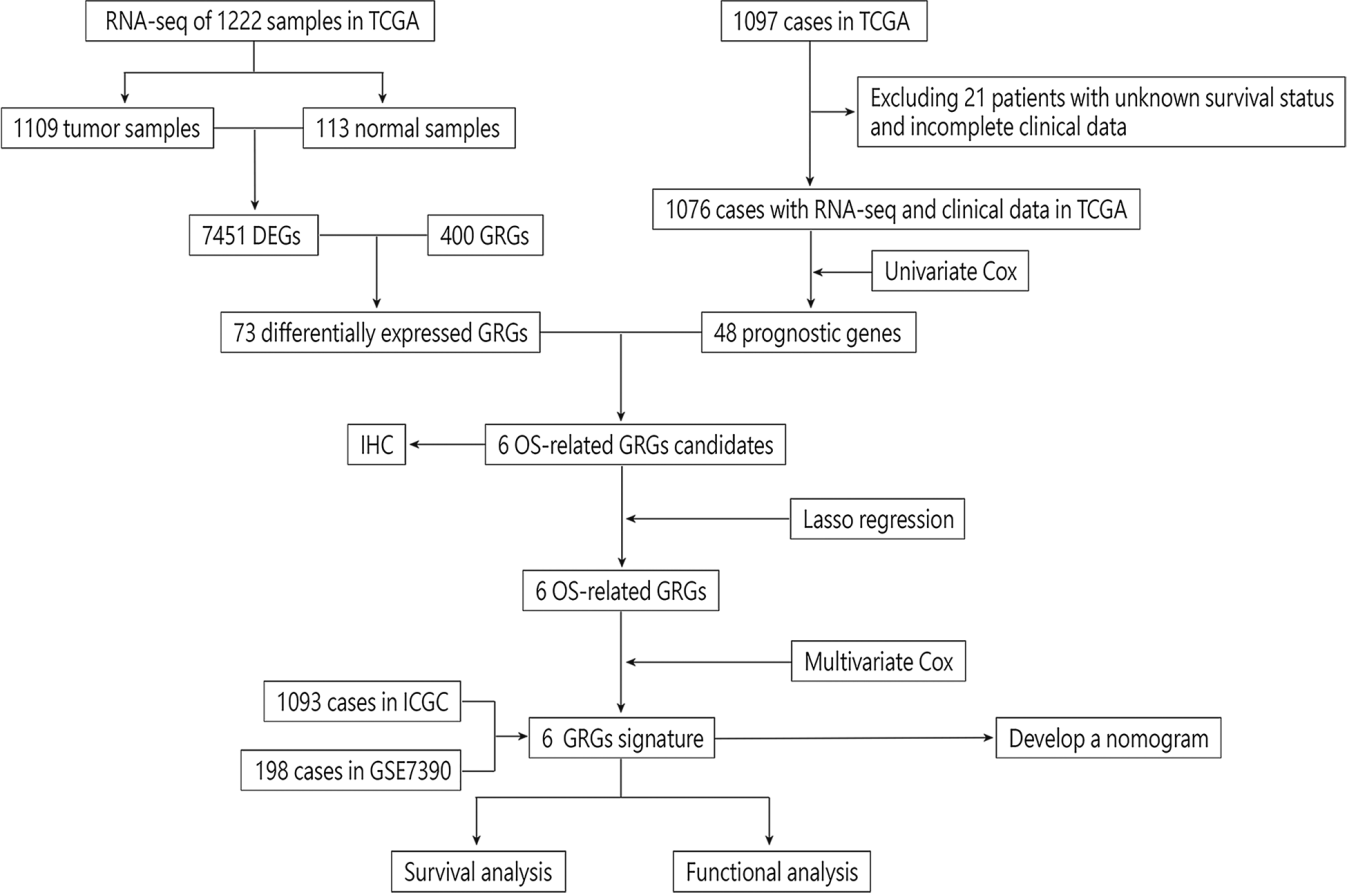
Flow chart of a GRG signature in BRCA patients.

**Figure 2:**
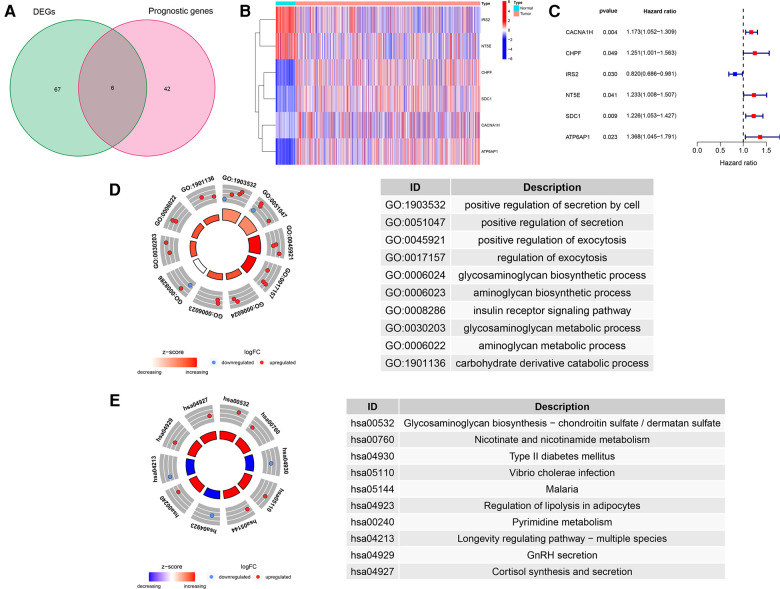
Identification of GRG candidates in TCGA cohort. (**A**) Venn diagrams of DEGs and Prognostic genes. (**B**) The expression pattern of GRG candidates. (**C**) Univariate Cox regression analysis of GRG candidates. (**D**) GO enrichment of GRG candidates. (**E**) KEGG enrichment of GRG candidates.

We draw the flow chart to demonstrate our research ideas more clearly (Figure 1). These 73 DEGs related to glucose metabolism were obtained between 1,109 tumor tissues and 113 normal tissues (FDR < 0.05, |Log FC|> 1). After univariate Cox regression analysis, 48 prognostic genes were found which were significantly correlated with the OS of BRCA patients (*P* < 0.05). Taking the intersection of the DEGs and the Prognostic genes in the TCGA training cohort, we discovered the six GRG candidates (CACNA1H, CHPF, IRS2, NT5E, SDC1 and ATP6AP1) and included in subsequent analysis, as shown in Figure 2A.

We detected significant downregulation of IRS2 and NT5E in tumor tissues, whereas CACNA1H, CHPF, SDC1 and ATP6AP1 were highly expressed in tumor tissues than in normal tissues (Figure 2B). Candidate GRGs were categorized into risk genes [Hazard Ratio (HR) > 1] and protective genes (0 < HR < 1). Only IRS2 had a protective effect (HR = 0.820), whereas CACNA1H, CHPF, NT5E, SDC1 and ATP6AP1 all had risk effects (HR > 1) (Figure 2C). Moreover, we performed GO and KEGG enrichment analyses to verify whether the candidate genes are involved in glycometabolism. It was determined that the GO term was related to the glycosaminoglycan biosynthetic process and the glycosaminoglycan metabolic process, and that the KEGG term was related to Glycosaminoglycan biosynthesis-chondroitin sulfate/dermatan sulfate (Figures 2D,E, Supplementary Table S1).

### Construction and evaluation of a GRG signature

**Figure 3:**
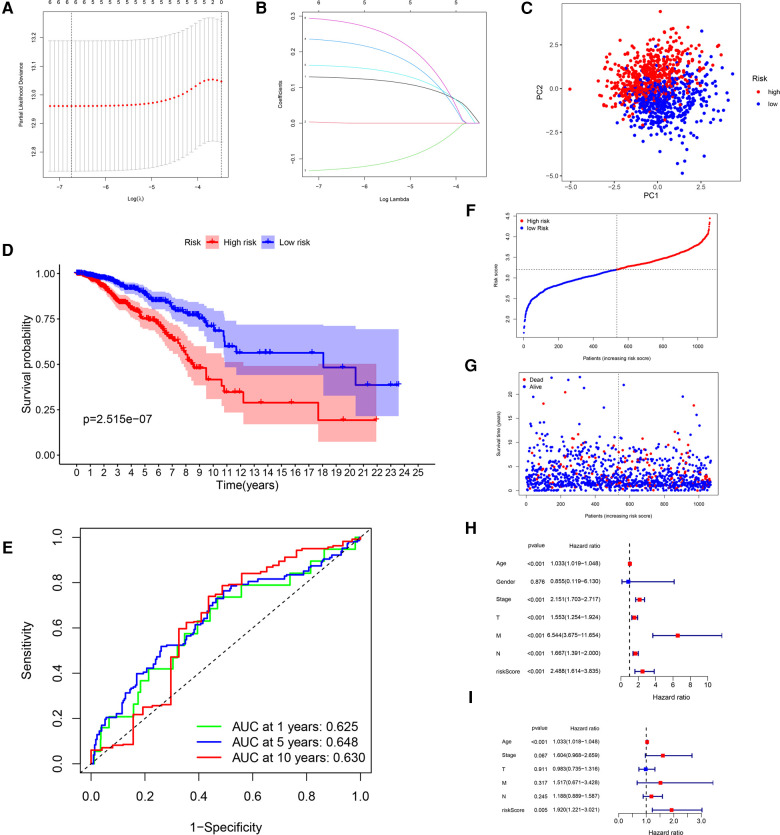
Construction of a GRG signature in TCGA cohort. (**A**) Selection of the optimal GRG candidates in the LASSO analysis. (**B**) LASSO coefficients of the optimal GRG candidates. (**C**) PCA based on risk score of GRGs. (**D**) Kaplan–Meier survival curves between high-risk and low-risk groups. (**E**) The ROC curves of 1-, 5- and 10-years OS. (**F,G**) Survival status of each patient. (**H,I**) Univariate and multivariate Cox regression analyses.

After LASSO algorithm to minimize the risk of overfitting, 6 GRGs were reserved, a GRG signature based on CACNA1H, CHPF, IRS2, NT5E, SDC1 and ATP6AP1 was established to evaluate the prognosis of each patient using multivariate Cox regression analysis (Figures 3A,B). The formula for calculating risk score was as follows: $\mathrm{Risk}\;\mathrm{score}=\sum_{i=1}^{n}\mathrm{Coef}\;*\;\mathrm{Exp}$ = (0.1281 × Exp CACNA1H) + (0.0016 × Exp CHPF) + (−0.1299 × Exp IRS2) + (0.2311 × Exp NT5E) + (0.1604 × Exp SDC1) + (0.2888 × Exp ATP6AP1).

In accordance with the median risk score, 1,076 BRCA patients, 6 patients were deleted for missing candidate genes, were divided into high-risk group (*N* = 535) and low-risk group (*N* = 535) in the risk prognostic model. The PCA confirmed that the risk score could be grouped significantly (Figure 3C). The Kaplan–Meier curves showed that patients in the low-risk group had a better OS than patients in the high-risk group in TCGA training cohort (*P* = 2.515 × 10^−7^; Figure 3D). The ROC curves were used to evaluate the prognostic accuracy of the GRG signature, the AUCs of the training cohort for predicting 1-, 5- and 10-years OS for breast cancer were 0.625, 0.648, 0.630 (Figure 3E). The survival status of the patients was shown in Figures 3F,G. As the risk score of GRG candidates increased, the mortality rate also increased, and the life expectancy in the high-risk group was shorter than in the low-risk group. The results of univariate and multivariate Cox regression analyses documented that the risk score was significantly associated with the OS (*P* < 0.05; Table 3; Figures 3H,I), indicating that GRG risk score may be more accurate than other clinical variables and used to analyse prognosis of BRCA patients.

**Table 3:** Univariate and multivariate Cox regression analyses for each clinical feature (TCGA cohort).

Clinical feature	Univariate analysis			Multivariate analysis		
	HR	95% CI of HR	*P* value	HR	95% CI of HR	*P* value
Age	1.0335	1.0190–1.0482	5.02 × 10^−6^	1.0327	1.0178–1.0478	1.44 × 10^−5^
Gender	0.8546	0.1192–6.1295	0.8758	–	–	–
Stage	2.1507	1.7026–2.7166	1.32 × 10^−10^	1.6044	0.9680–2.6591	0.0667
T	1.5531	1.2536–1.9241	5.62 × 10^−5^	0.9835	0.7349–1.3161	0.9107
M	6.5438	3.6745–11.6537	1.77 × 10^−10^	1.5169	0.6712–3.4280	0.3165
N	1.6674	1.3905–1.9995	3.43 × 10^−8^	1.1877	0.8886–1.5874	0.2452
Risk Score	2.4876	1.6136–3.8350	3.69 × 10^−5^	1.9201	1.2206–3.0206	0.0048

Abbreviations: T, Tumor; N, Node (regional lymph node); M, Metastasis; HR, Hazard ratio, 95% CI, 95% confidence interval.

### Validation of the GRG signature

**Figure 4:**
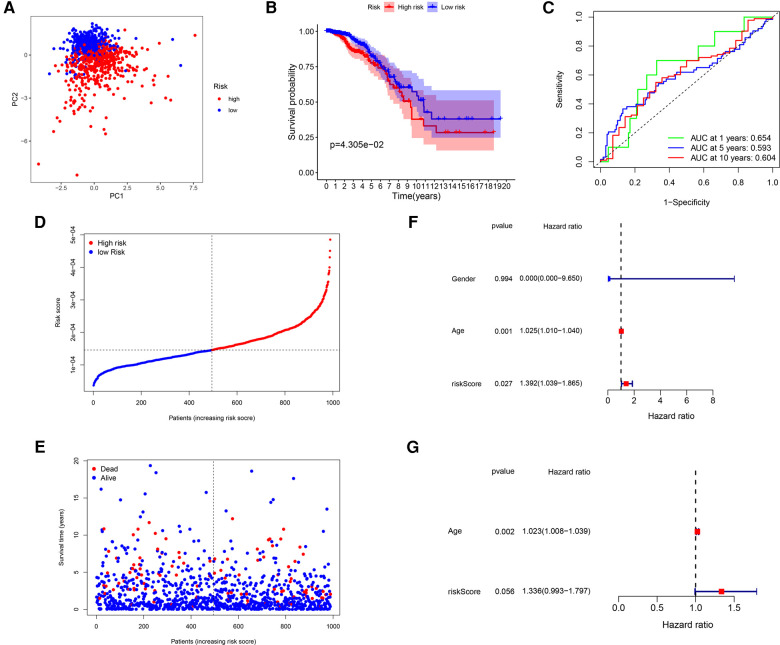
Validation of the GRG signature in ICGC cohort. (**A**) PCA based on risk score of GRGs. (**B**) Kaplan–Meier survival curves between high-risk and low-risk groups. (**C**) The ROC curves of 1-, 5- and 10-years OS. (**D,E**) Survival status of each patient. (**F,G**) Univariate and multivariate Cox regression analyses.

After that, we validated the predictive ability of the GRG signature in the ICGC cohort. The same formula as TCGA cohort was used to calculate the risk score of each patient and group them by the median risk score derived from the dataset, and a separate high-risk group and a low-risk group could be clearly identified by PCA (Figure 4A). There were better outcomes for low-risk patients as compared to high-risk patients in Kaplan–Meier curves (*P* = 4.305 × 10^−2^; Figure 4B), which the AUCs of predicting 1-, 5- and 10-years OS were 0.654, 0.593, 0.604 in the ICGC validated cohort (Figure 4C). Just as with the training cohort, the number of deaths increased as patients' risk scores increased (Figures 4D,E), and the risk score could be used as an independent prognostic indicator (Table 4; Figure 4F,G). In addition, we also performed validation in the GSE7390 cohort and obtained consistent results with the training cohort (Supplementary Figure S1).

**Table 4:** Univariate and multivariate Cox regression analyses for each clinical feature (ICGC cohort).

Clinical feature	Univariate analysis			Multivariate analysis		
	HR	95% CI of HR	*P* value	HR	95% CI of HR	*P* value
Gender	2.99 × 10^−7^	0–9.65	0.9944	–	–	–
Age	1.0246	1.0096–1.0398	0.0012	1.0233	1.0082–1.0385	0.0024
Risk score	1.3922	1.0392–1.8649	0.0266	1.3356	0.9929–1.7967	0.0558

### Relationship between risk score and clinical features

**Figure 5:**
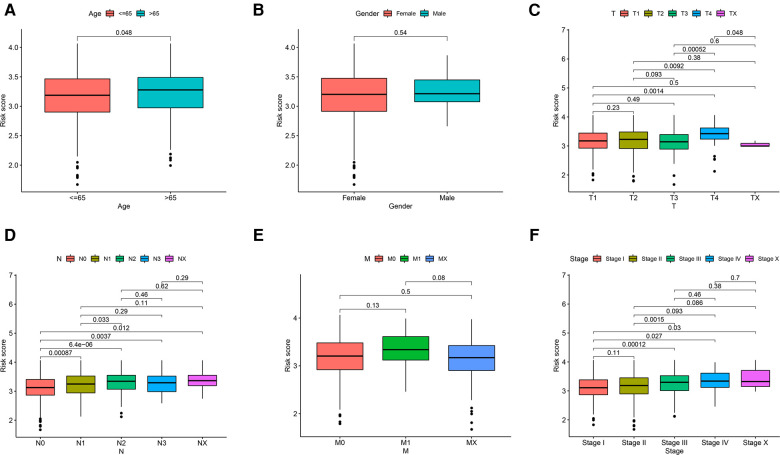
Relationship of risk score and clinicopathological features, including (**A**) age, (**B**) gender, (**C**) AJCC-T, (**D**) AJCC-N, (**E**) AJCC-M and (**F**) stage.

Even though gender and AJCC-M did not significantly affect risk scores (Figures 5B,E), risk scores were correlated with age, AJCC-T, AJCC-N and stage. Age-adjusted risk scores of breast cancer were slightly higher for patients over 65 than for those under 65 (*P* = 0.048, Figure 5A). We see no significant change in risk scores from T1 to T3, but T4 is significantly higher than T3 (*P* = 0.00052, Figure 5C). The higher risk scores were correlated with higher AJCC-N classification from N0 to N2 (*P* < 0.05; Figure 5D). The patients who entered an advanced stage (stage III–IV) had a higher risk score than those who were in the early stage (stage I–II) (*P* = 0.0015; Figure 5F).

### Analysis of tumor microenvironment and GRG signature

**Figure 6:**
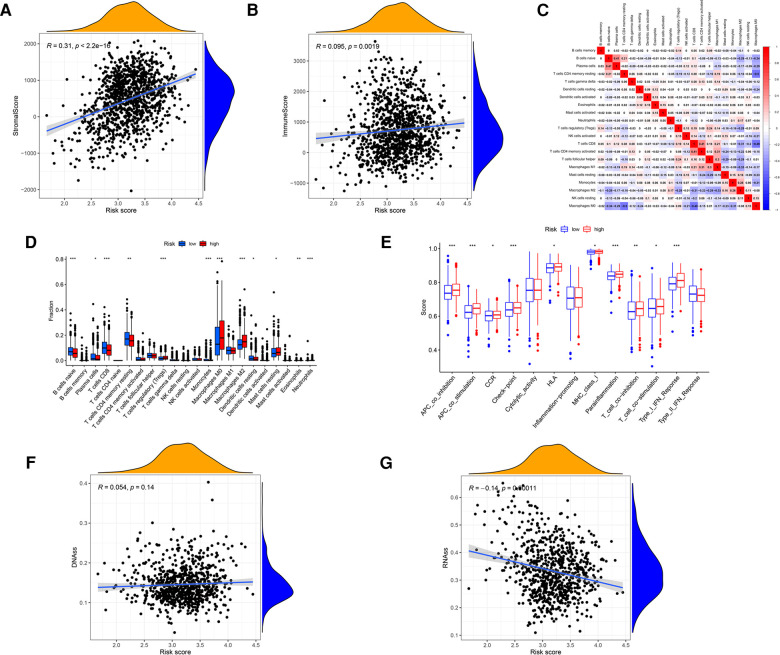
Analysis of the GRG signature in tumor microenvironment, including (**A**) stromal cells, (**B**) immune cells, (**C**) correlations among the immune cells, (**D**) 22 kinds of tumor-infiltrating immune cells, (**E**) 13 kinds of immune related function, (**F**) DNAss and (**G**) RNAss.

This study investigated the association between glucose metabolism scores and TME properties. The stromal (*P* < 2.2 × 10^−16^; Figure 6A) and immune (*P* = 0.0019; Figure 6B) scores of high-risk groups were significantly higher than those of low-risk groups, defined as the characteristic of “hot tumor” (34), indicating that tumor immune activity was stronger in high-risk patients than in low-risk patients. Correlations among the immune cell types are plotted in Figure 6C. As presented in Figure 6D, infiltrating proportions of regulatory T cells (Tregs), M0 Macrophages, M2 Macrophages and resting Mast cells were apparently higher in high-risk patients, while infiltrating abundance of naive B cells, Plasma cells, CD8^+^ T cells, resting memory CD4^+^ T cells and resting Dendritic cells were significantly increased in low-risk patients. Next, we explore how immune-related functions differ between risk groups, the enrichment scores of immune-related functions including APC-co-inhibition, APC-co-stimulation, check-point, Parainflammation, T-cell-co-inhibition, T-cell-co-stimulation and Type-1-IFN-Reponse in the high-risk group were markedly higher than these in the low-risk group (Figure 6E). Additionally, we found no correlation between risk score and DNAss (Figure 6F), yet an inverse correlation with RNAss (Figure 6G).

### Comparison of antineoplastic therapy between the low-risk and high-risk groups

**Figure 7:**
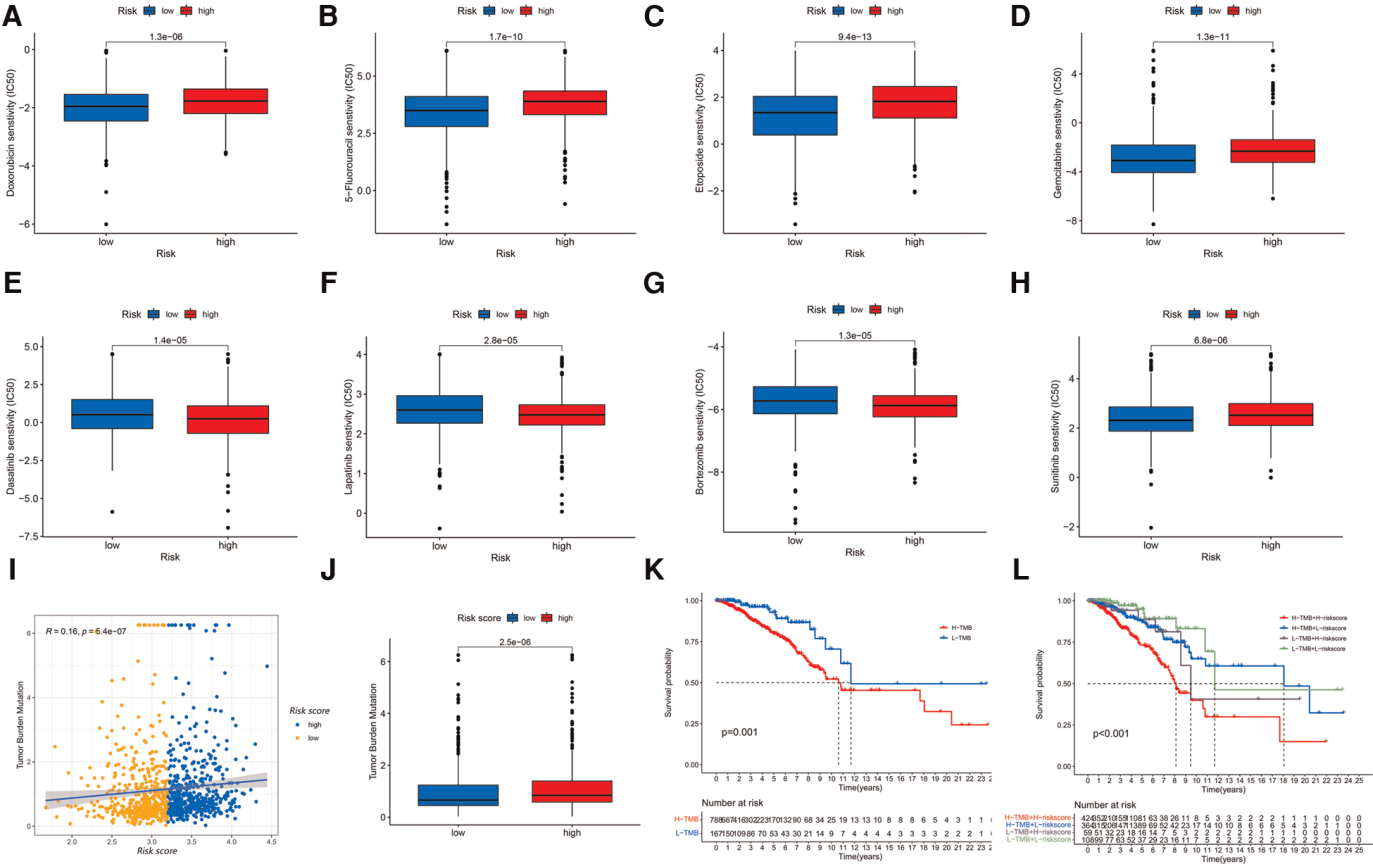
The GRG signature in the role of antineoplastic therapy, including (**A**) doxorubicin, (**B**) 5-fluorouracil, (**C**) etoposide, (**D**) gemcitabine, (**E**) dasatinib, (**F**) lapatinib, (**G**) bortezomib and (**H**) sunitinib. (**I,J**) Correlations and difference of TMB between high-risk and low-risk groups. (**K**) Survival probability for patients between high-TMB and low-TMB groups. (**L**) Survival probability for patients combining TMB with risk score.

As the risk score was associated with poor prognosis, we explored the relationship between the risk score and drug sensitivity. In chemotherapy drugs, low-risk score samples were more sensitive to Doxorubicin, 5-Fluorouracil, Etoposide and Gemcitabine (Figures 7A–D). In targeted therapies, high-risk samples were more sensitive to Dasatinib, Lapatinib and Bortezomib, while low-risk samples were sensitive to Sunitinib (Figures 7E–H). Tumor mutation burden (TMB) has been recognized as a marker for identifying malignant patients who may benefit from immunotherapy. We found that there was a positive correlation between TMB and risk score, and the high-risk group had higher levels of TMB (Figures 7I,J), suggesting a better effect of immunotherapy. The low-TMB group had a higher survival rate than the high-TMB group (*P* = 0.001, Figure 7K). In addition, patients in the high-risk group with high-TMB were at a disadvantage in survival (*P* < 0.001, Figure 7L).

### Screening of risk differential and hub genes

**Figure 8:**
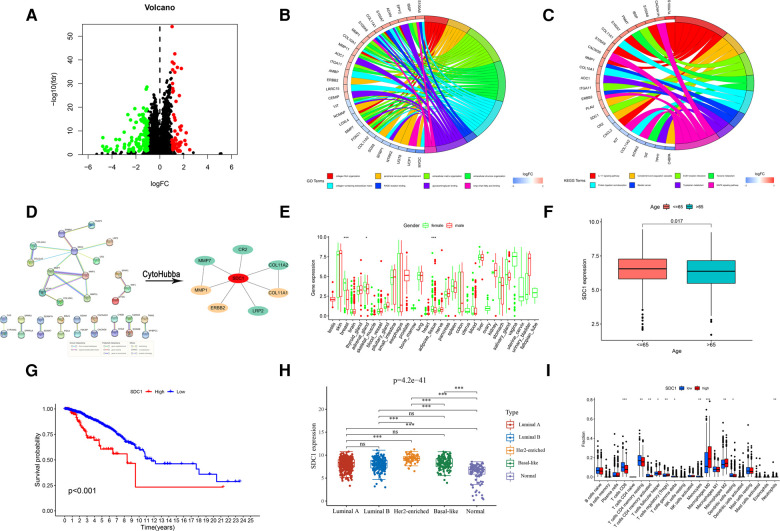
Screening and analysis of RDEGs. (**A**) Screening of RDEGs. (**B**) GO enrichment of RDEGs. (**C**) KEGG enrichment of RDEGs. (**D**) PPI of RDEGs and identification of Hub gene. Expression difference of Hub gene, including (**E**) Gender, (**F**) Age and (**H**) different types of breast cancer. (**G**) OS analysis of Hub gene. (**I**) Immune infiltration of Hub gene.

A total of 175 risk differential expression genes (RDEGs) were identified between high-risk and low-risk groups (FDR < 0.05, |Log FC| > 1), of which were 55 up-regulated and 120 down-regulated (Figure 8A). GO and KEGG analysis showed that these 175 RDEGs were significantly abundant in various roles. For Go analysis, the top eight significantly enriched terms were collagen fibril organization, peripheral nervous system development, extracellular matrix organization, extracellular structure organization, collagen-containing extracellular matrix, RAGE receptor binding, glycosaminoglycan binding and long-chain fatty acid binding (Figure 8B, Supplementary Table S2). The enriched KEGG items were revealed in Figure 8C, including IL-17 signaling pathway, Complement and coagulation cascades, ECM-receptor interaction, Tyrosine metabolism, Protein digestion and absorption, Tryptophan metabolism and MAPK signaling pathway. Cytoscape software used Degree algorithm to identify Hub gene (SDC1) from the PPI network established by String database (Figure 8D, Supplementary Table S3). We found a gender difference in the expression of SDC1 in breast, adrenal_gland and adipose_tissue, which was slightly higher in females than in males (Figure 8E). In addition, SDC1 was more strongly expressed in patients under 65 years of age than in patients over 65 years of age in BRCA patients (Figure 8F), and the OS of high-SDC1was poorer than that of low-SDC1 (Figure 8G). We also found that SDC1 expression varies widely among different types of breast cancer, with Her2-enriched exhibiting among the highest levels of expression (Figure 8H). There were more CD8^+^ T cells in the low-SDC1 group, and more M2 macrophages in the high-SDC1 group (Figure 8I).

### Establishment and validation of a predictive nomogram

**Figure 9:**
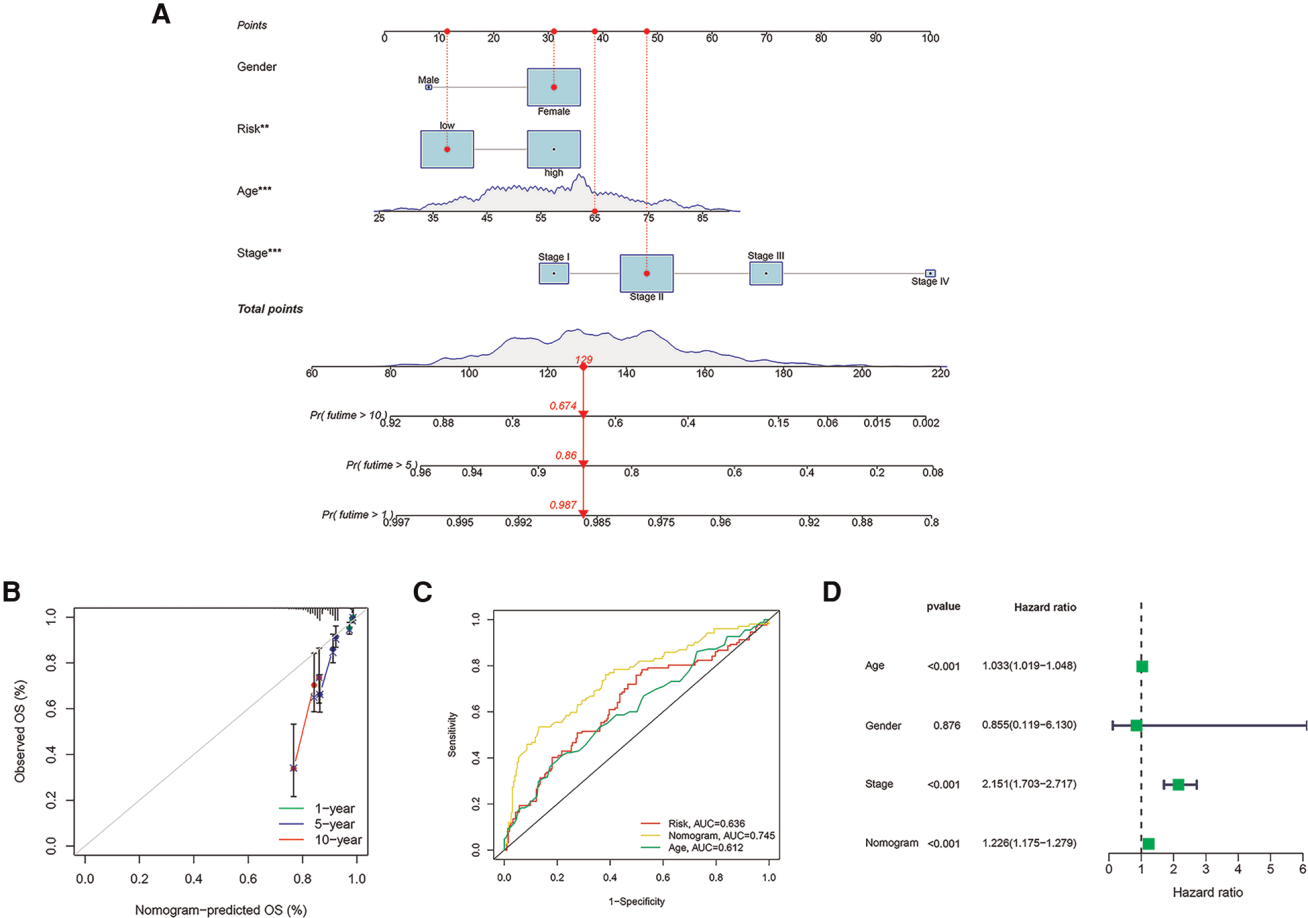
Establishment of a nomogram based on GRG signature. (**A**) The nomogram integrating GRG risk score, gender, age and stage. (**B**) The calibration curves for the probability of 1-, 5- and 10- years OS. (**C**) The ROC curves of nomogram, risk score and other clinicopathological characteristics. (**D**) Cox regression analysis of nomogram.

Using the TCGA cohort of 1,076 patients with complete clinical information, a prognostic nomogram was established to predict OS of 1-, 5- and 10- years based on GRG signature and independent prognostic parameters (Figure 9A). The calibration curves showed agreement between predicted and observed OS (Figure 9B). The ROC curves indicated that the risk score was valid in OS prediction, yet the nomogram showed a greater advantage (AUC_Risk _= 0.636; AUC_Nomogram _= 0.745; Figure 9C). Consistently, Cox regression analysis also showed that the predictive ability of nomogram is admirable (Figure 9D).

### Expression validation of GRG candidates in protein level

**Figure 10:**
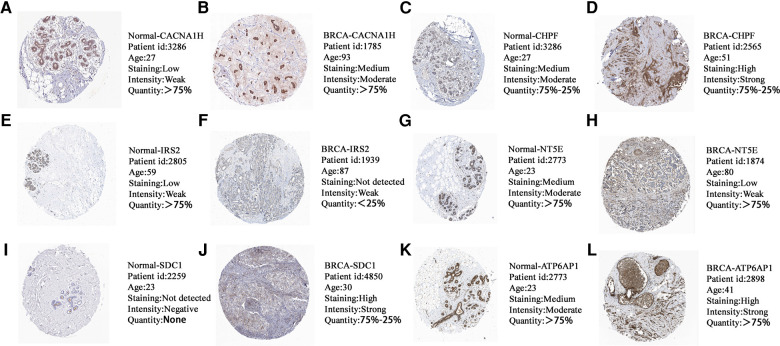
The protein expression levels of (**A,B**) CACNA1H, (**C,D**) CHPF, (**E,F**) IRS2, (**G,H**) NT5E, (**I,J**) SDC1 and (**K,L**) ATP6AP1 in HPA database based on immunohistochemistry.

The Human Protein Atlas (HPA) is a well-known database for detecting protein expression in various solid cancers (35). We observed the immunohistochemistry of GRG candidates through the HPA database to confirm the protein expression of them in normal and breast cancer tissues. We found that the protein levels of CACNA1H, CHPF, SDC1 and ATP6AP1 were higher in breast cancer tissues compared with normal tissues, while the expression levels of IRS2 and NT5E were comparatively lower in breast cancer tissues (Figure 10), and the expression of them is consistent with mRNA level (Figure 2B).

## Discussion

Currently, few studies have focused on the expression patterns of GRGs and their role in predicting breast cancer survival (36). Recent studies have shown that more and more mRNAs have been identified as biomarkers of tumor progression or prognosis, and the clinical significance of these biomarkers has been confirmed (37–39). In our study, we examined the relationship between GRGs expression level and survival in BRCA patients. We developed a novel GRG signature of six GRGs, including CACNA1H, CHPF, IRS2, NT5E, SDC1 and ATP6AP1, to predict survival and guide individual therapy in breast cancer. Univariate and multivariate Cox regression analyses ensured the prognostic value of the GRG signature.

Consistent with Prof. Pera (40), we found that CACNA1H had high expression and high mutation rate in breast cancer. CACNA1H is a T-type calcium channel gene whose mutation can lead to hyperpolarization, resulting in channel activation and massive calcium influx (41). The pathologic opening of calcium channels leads to the continuous influx of calcium ions, which affects the division and proliferation of normal cells and causes the carcinogenesis of cells. CHPF is a common glycosyltransferase involved in the production of Chondroitin Sulfate in organisms (42). CHPF is highly expressed in lung adenocarcinoma and can promote tumor cell growth, invasion and metastasis, and inhibit tumor cell apoptosis (43). More specifically, the role of CHPF in breast cancer is to promote proliferation, invasion and migration (44). IRS2 is an insulin-like growth factor-1 receptor (IGF-1R) and insulin receptor signaling transmitter, which may be involved in the PI3K-AKT pathway, leading to the occurrence and progression of malignant tumors and inhibition of apoptosis (45, 46). It is worth mentioning that IRS2 is also closely related to the invasion and metastasis of malignant cells (47). Therefore, IRS2 is expected to be a potential target for antitumor therapy. CD73, encoded by NT5E, is a glycosyl phosphatidylinositol-anchored cell surface protein, also known as ECTO-5'-Nucleotide Enzyme (NT5E), which regulates immunosuppressive adenosine production and is an emerging checkpoint in immunotherapy (48). Many studies have shown an association between increased CD73 expression and poor prognosis in patients (49). Syndecan-1 (SDC1/CD138) is a key cell surface adhesion molecule that is essential for maintaining cell morphology and interaction with the surrounding microenvironment (50). Researchers have found that high expression of SDC1 was significantly associated with adverse clinical outcomes of cancer (51). ATP6AP1 represents ATPase H^+^ transporting accessory protein 1 (52), and mutations in the protein lead to an abnormal glycosylation process (53). Wang et al. found that the expression of ATP6AP1 was negatively correlated with CD8^+^ T and B cell infiltration, ATP6AP1 could induce immunosuppression and immune escape, and may worsen the prognosis of BRCA patients by regulating immune infiltration (54). These results indicate that glycometabolism-related genes will be promising targets in breast cancer.

Glucose metabolism may be involved in immune cell infiltration in the tumor microenvironment, which contributes to immunotherapy response. Hyperactivation of glucose metabolism can lead to an acidic microenvironment, which affects the function of immune cells and creates an immune microenvironment conducive to tumor cell survival (55). As we found, although BRCA patients in the high-risk group had higher proportions of immune cells, including Tregs and M2 Macrophages, they had poorer overall survival. The large amount of lactate produced by excessive glucose metabolism can promote the differentiation of tumor-associated macrophages into M2 subtype which against the anti-tumor immunity in the tumor microenvironment (56, 57), and may explain the above conclusion. In patients with low-risk group, a higher infiltrating abundance of CD8^+^ T cells and resting memory CD4^+^ T cells were found, which contributed to the anti-tumor immunity and were positively correlated with prognosis (58, 59). Stromal cells as an essential component of the tumor microenvironment could secrete CCL-2, a chemokine that promotes tumor cell migration, proliferation and angiogenesis (60).

Besides, we investigated the sensitivity of chemotherapy and targeted drugs in risk groups. The low-risk group was more sensitive to chemotherapy, while the high-risk group was more sensitive to targeted therapy, such as Lapatinib. Lapatinib was first approved by the FDA as a tyrosine kinase inhibitor (TKI) in 2007 for the treatment of BRCA patients who were HER2-positive/ER-negative/PR-negative (61, 62). Previous study had shown that patients with a high level of TMB had a better response to immunotherapy (29). We found higher TMB expression in the high-risk patients, which indicated that high-risk score patients might benefit more from immunotherapy. Therefore, the GRG signature could guide clinicians to choose more beneficial treatment options for BRCA patients.

Since there are significant differences between the low-risk and high-risk groups, we further examined different genes between the two groups. SDC1 was found to be the central gene of RDEGs, and patients in the low-SDC1 group with more CD8^+^ T cells had a better OS. Among all patients with breast cancer, about 20%–30% will show positive Her-2, which is a warning signal. Positive Her-2 indicates that it is a highly invasive cancer and more likely to relapse and metastasis (63). We found higher SDC1 in patients with Her2-enriched. SDC-1 is a membrane-anchored protein polysaccharide expressed on the basolateral surface of epithelial cells, which is abnormally induced in breast cancer stromal fibroblasts and plays a key role in tumor proliferation (64). In addition, SDC-1 acts as a receptor on the cell surface to form a complex with integrin and receptor tyrosine kinase (RTK) to regulate proliferation and migration (65). Moreover, overexpression of SDC1 resulted in increased angiogenesis promoters (66), including FGF2 and VEGF, and the anti-SDC1 antibody 46F2SIP was confirmed to effectively inhibit angiogenesis and induce vascular normalization (67). It was found that the reduction of SDC1 arrested cells from S phase to G1 phase, slowed cell cycle progression and inhibited cell proliferation (68). Therefore, SDC1 may be a suitable targeted therapeutic molecule for BRCA patients with various types.

Previous research has shown that these biomarkers are still not enough to predict the prognosis of patients independently. In particular, the level of single gene expression may be affected by multiple factors and they cannot be used as reliable and independent prognostic indicators (18). Therefore, a prognostic signature consisting of multiple related genes combined with the predictive effect of each gene, which is used to improve the predictive ability (69). The signature is much more accurate in assessing the prognosis of patients with breast cancer than using a single biomarker, so it will be widely used. However, we can only use OS to evaluate the prognosis of patients due to the lack of metastasis and recurrence information of patients in TCGA, ICGC and GSE7390 cohorts, which is one of the limitations of our study. In addition, we will further confirm our findings in cell and tissue experiments, and explore their potential mechanisms in the development of breast cancer.

## Conclusion

In summary, the GRG candidates comprising CACNA1H, CHPF, IRS2, NT5E, SDC1 and ATP6AP1 were identified and incorporated into a novel risk model to predict prognosis. Besides, the risk score of GRG signature can not only characterize patients' clinicopathological features, but also predict sensitivity of chemotherapy, targeted therapy and immunotherapy. We believe that the novel GRG signature will promote individualized treatments and improve OS of BRCA patients.

## Data Availability

The original contributions presented in the study are included in the article/**Supplementary Material**, further inquiries can be directed to the corresponding author/s.
