# Supplementary figures and images for: Characterization of glucose metabolism in breast cancer to guide clinical therapy
Source: Front Surg. 2022 Sep 19;9:973410. doi: 10.3389/fsurg.2022.973410 (PMC9580338; doi:10.3389/fsurg.2022.973410)

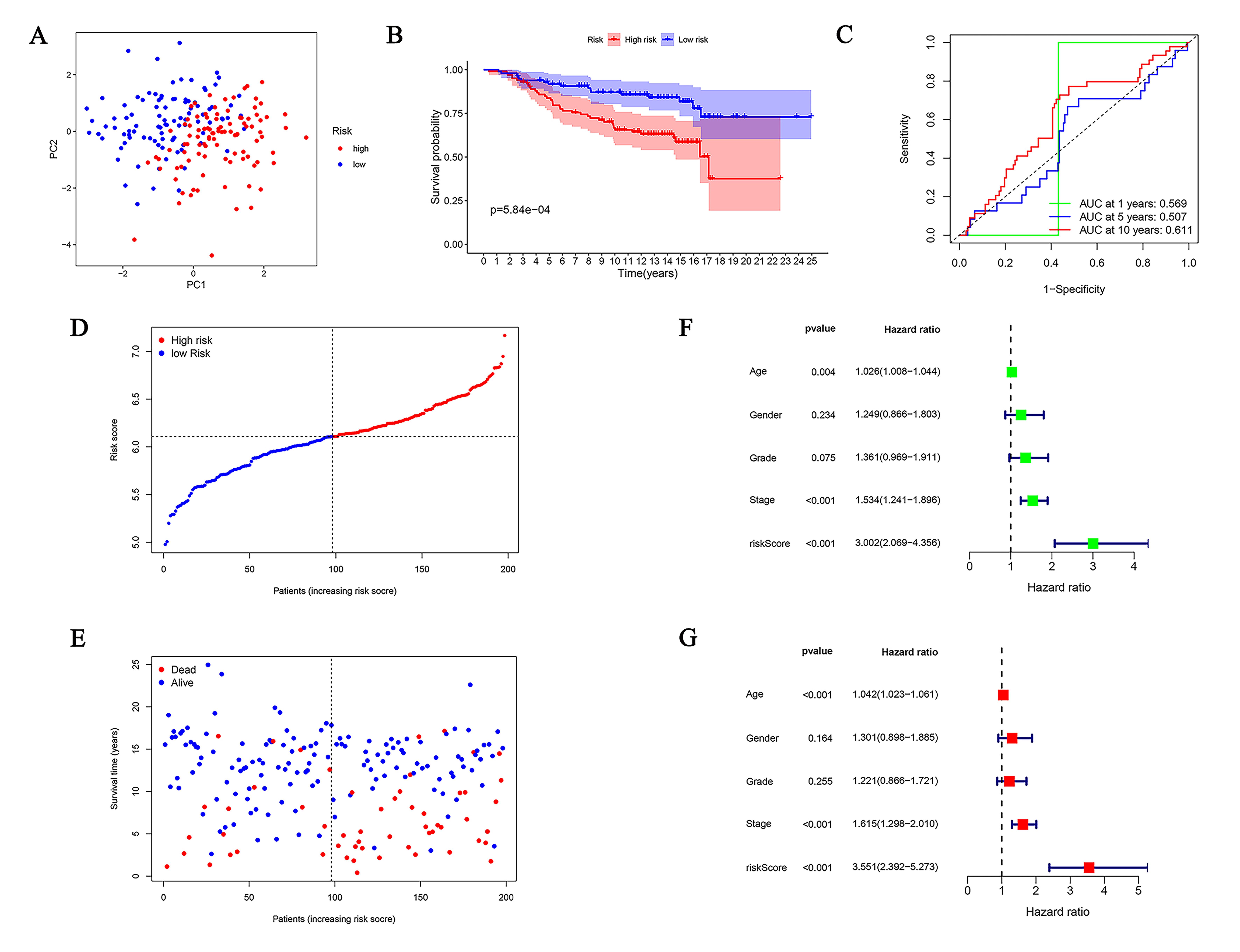

Supplement: Supplementary file 1 [file Image1.jpeg]
